# Predictive Coding: A Possible Explanation of Filling-In at the Blind Spot

**DOI:** 10.1371/journal.pone.0151194

**Published:** 2016-03-09

**Authors:** Rajani Raman, Sandip Sarkar

**Affiliations:** Applied Nuclear Physics Division, Saha Institute of Nuclear Physics, Kolkata, India; McGill University, CANADA

## Abstract

Filling-in at the blind spot is a perceptual phenomenon in which the visual system fills the informational void, which arises due to the absence of retinal input corresponding to the optic disc, with surrounding visual attributes. It is known that during filling-in, nonlinear neural responses are observed in the early visual area that correlates with the perception, but the knowledge of underlying neural mechanism for filling-in at the blind spot is far from complete. In this work, we attempted to present a fresh perspective on the computational mechanism of filling-in process in the framework of hierarchical predictive coding, which provides a functional explanation for a range of neural responses in the cortex. We simulated a three-level hierarchical network and observe its response while stimulating the network with different bar stimulus across the blind spot. We find that the predictive-estimator neurons that represent blind spot in primary visual cortex exhibit elevated non-linear response when the bar stimulated both sides of the blind spot. Using generative model, we also show that these responses represent the filling-in completion. All these results are consistent with the finding of psychophysical and physiological studies. In this study, we also demonstrate that the tolerance in filling-in qualitatively matches with the experimental findings related to non-aligned bars. We discuss this phenomenon in the predictive coding paradigm and show that all our results could be explained by taking into account the efficient coding of natural images along with feedback and feed-forward connections that allow priors and predictions to co-evolve to arrive at the best prediction. These results suggest that the filling-in process could be a manifestation of the general computational principle of hierarchical predictive coding of natural images.

## Introduction

Filling-in at the blind spot is one of the examples of how brain interpolates the informational void due to the deficit of visual input from the retina. Because of the absence of photoreceptors at optic disc, the retina is unable to send the corresponding signal to the brain and thereby, hides some portion of the visual field. The concealed visual field is known as the blind spot. However, we never notice any odd patch in our visual field, even in monocular vision, but rather we see the complete scene; filled up in accordance with the surrounding visual attributes [1]. This completion is known as perceptual filling-in or simply filling-in. In addition to the blind spot, filling-in also occurs in other visual input deficit conditions, e.g. filling-in at the artificial and natural retinal scotoma [2, 3]. In addition to the deficit of input, filling-in also occurs in visual illusions such as Neon color spreading, Craik-O’Brien-Cornsweet illusion, Kanizsa shapes, etc. and steady fixation condition like Troxler effect (for review see [4]).

**Fig 1:**
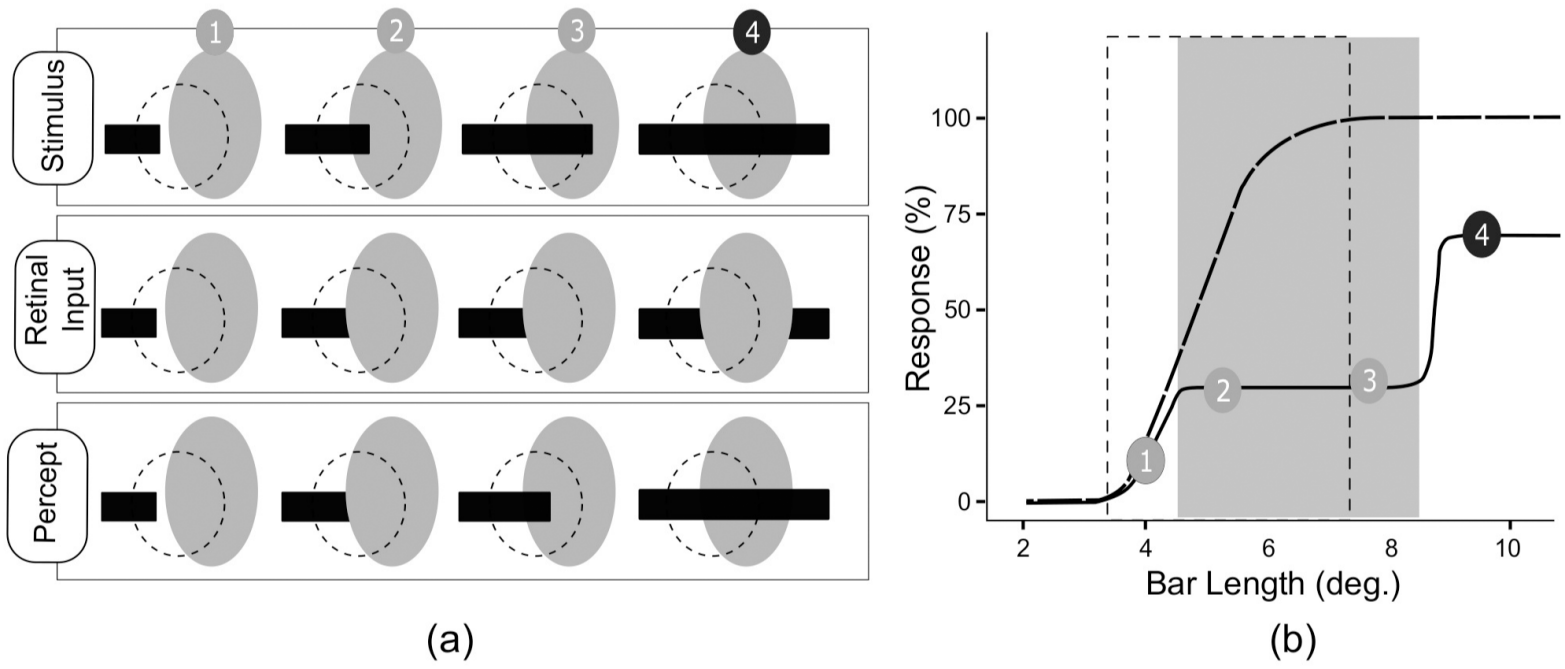
Schematic illustration of bar completion experiment (adopted from Matsumoto and Komatsu [4]). (a) The gray oval area represents the blind spot, whereas the dashed circle represents the receptive field of a neuron. The actual stimulus, the corresponding retinal input and percept at position 1,2,3 and 4 are shown. One end of the bar stimuli was kept fixed outside the BS region, and the other end was free to drift across the blind spot. (b) The response of a typical neuron in BS region at the deep layer of primary visual cortex is presented. The gray rectangle indicates the blind spot and the dotted rectangular area represent the receptive field of the typical neuron. The solid line is the response obtained through the eye connected to the blind spot (BS eye) under review, and the dotted line is the response of the same neuron obtained through the fellow eye. While the drifting end of the bar was inside the blind spot the perception of the bar was of a short isolated bar and corresponding neural responses were low and constant. However, the moment bar end crossed the blind spot, the neural response elevated rapidly and completion of the bar was perceived. These elevated response exhibit nonlinearity; the response to the long bar that stimulate simultaneously the both sides of the blind spot was larger than the sum of responses to the stimuli presented on either side of blind spot separately.

Many psychophysical and physiological studies have been performed to gain insight into the neural mechanism of perceptual completion at the blind spot. These studies suggest that the filling-in is an active process: some neural process is involved and mainly take place in the early visual cortex [5–7]. For example, studies on monkeys show that perceptually correlated neural activities are evoked in the deep layer of primary visual cortex, in the region that retinotopically corresponds to the blind spot (BS) region, when filling-in completion occurs [5, 6]. In another experiment, Matsumoto and Komatsu [7] showed that some neuron in BS region in deep layer of primary visual cortex (BS neurons), which possess larger receptive fields that extend beyond the blind spot, exhibits non-linear elevated response when a long moving bar cross over the blind spot and perceptual completion occurs (See Fig 1).

Although some attempts have been made to understand the computational mechanism of completion of illusory contour and surface [8–12], little work has been devoted to the study of computational mechanism of filling-in completion at the blind spot. Recent studies [13] have suggested the computational mechanism of completion of the bar in terms of a complex interaction of velocity-dependent pathways in the visual cortex under the framework of regularization theory. However, the fitness of this suggestion in the context of a general coding principle of the visual cortex is not clear. Here in this study, we suggest the filling-in completion at the blind spot naturally follows from the computational principle of Hierarchical predictive coding (HPC)of natural images, which has, recently, gained growing support as the general coding principle of visual cortex [14–24] (For an excellent review see [25]).

The root of Hierarchical predictive coding lies in the probabilistic hierarchical generative model and the efficient coding of natural images. In such probabilistic frameworks, the job of the visual system is to infer or estimate the properties of the world from signals coming from receptors [26–28]. In HPC framework, this job is hypothesized to be completed by concurrent prediction-correction mechanism along the hierarchy of the visual system. Accordingly, each higher visual area (say V2) attempt to predict response at its lower area (say V1) on the basis of the learned statistical regularities, and send that prediction signal to the lower area by feedback connection. In response to this top-down information, lower area sends a residual error signal to the higher area, by feed-forward connection, to correct the next prediction. This idea is based on the anatomical architecture of the visual system which is hierarchically organized and reciprocally connected [29]. Probabilistic generative model, in HPC framework, accounts for learning the statistical regularities found in natural images and generation of prediction of input based on that learning. Recently, several neuronal tuning properties in different visual areas such as the lateral geniculate nucleus (LGN), primary visual cortex (V1) and middle temporal level (MT) have been explained using this framework [17, 20, 21, 23]. For example, in his standard HPC model, Rao [14] suggested that the extra-classical properties of neurons in V1 could be understood in terms of the predictive-feedback signal from the secondary visual cortex (V2) which is made in a larger context and in the backdrop of learned statistical regularity of the natural Scene. We speculated that a similar mechanism could also explain the filling-in completion across the blind spot.

In this work, we have conducted simulation studies involving horizontal bars on three leveled (LGN-V1-V2) HPC model network having a blind spot which was emulated by removing the feed-forward (LGN-V1) connection. In our first investigation we have employed shifting bar stimuli as described in [7](See Fig 1), to study the properties of our model network and recorded the model predictive estimator neurons (PE neurons) at BS region in the V1. We found that these neurons exhibit similar non-linear response and represent the filling-in completion when bar crosses the blind spot. In another investigation, we presented two separate bar segments at the opposite end of the model blind spot to verify the tolerance of completion by varying the alignment of those segments. We found that the filling-in completion is best when the bars are perfectly aligned. The completion is visible for small orders of misalignment, but it fades out quickly with increasing misalignment. These results are consistent with the finding of psychophysical experiments [30, 31]and therefore, suggest that the filling-in process could naturally arise out of the computational principle of hierarchical predictive coding (HPC) of natural images.

## Methods

### Hierarchical Predictive coding of natural images

#### General Model and Network Architecture

**Fig 2:**
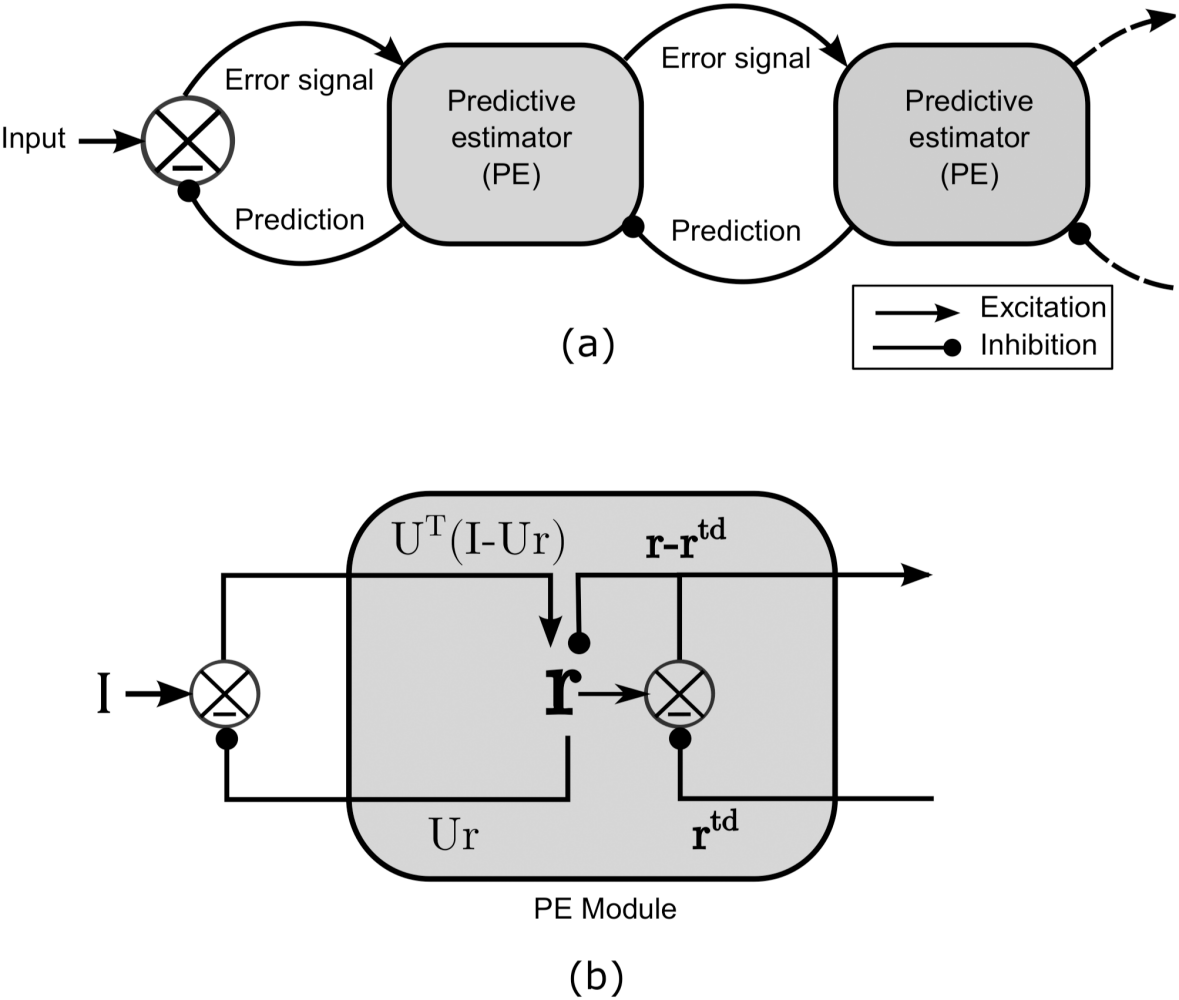
General HPC architecture (adopted from Rao. [14]). a) On arrival of input, predictive estimator module at each higher visual processing level makes the estimate and sends prediction signal to its next lower level by feedback connection and receives the corresponding prediction error by a feed-forward connection. The error signal is used by the predictive estimator module to correct the estimate for better prediction. b) General predictive estimator (PE) module constitutes of (i) neurons to represent the estimate of the input I by their response vector r by minimizing the bottom-up (**I** − *U***r**) and top-down (**r** − **r**^*td*^) error, (ii) feed-forward error carrying neurons has the efficacy matrix *U*, which encode the basis vectors their synaptic weights (or receptive fields), (iii) prediction *U***r** carrying neurons and (iv) top down error detecting neurons.

As discussed in the previous section, the problem of vision has been considered as an inference or an estimation problem; where an organism tries to estimate the hidden physical cause (object attributes such as shape, texture and luminance etc.) behind the generated image that organism receives as an input. In the HPC framework [14], it is assumed that the image generation in the outer world involves hierarchical, multilevel, spatial and temporal interactions between the physical causes. The goal of the visual system is, thus, to estimate (or internally represent) these multilevel hidden physical causes efficiently; which is accomplished by the visual system using recurrent prediction-correction mechanism along its hierarchy (See Fig 2a).

In this framework, on the arrival of an input, predictor estimator modules (PE module), at each visual processing level, generate the prediction (or estimate) on the basis of the learned statistical regularities of natural scenes. Each higher area (say V2) then sends the generated prediction to its immediate lower level (say V1) by feedback connections and in return receives the error signal, by feed-forward connections, which is used to correct the current estimate. An equilibrium state is achieved after the completion of few prediction-correction cycle; where the estimate matches the input signal. This optimum-estimate is regarded as a representation of the input at that level. The achieved optimum-estimate at different levels of network is depicted as a perception of the input image.

In general, a single PE module (See Fig 2b) consists of: (i) Predictive estimator neurons (PE neurons) which represent the estimate of current input signal **I** with response vector **r** (state vector), (ii) neurons, carrying prediction signal *U***r** (for the input **I**) to lower level by feed-back connections, whose synapse encode encoding efficacy matrix *U*, (iii) neurons, carrying feed-forward error signal (**I** − *U***r**) form lower level to higher level, whose synapses encoded rows of efficacy matrix *U*^*T*^, and (iv) error detecting neurons which carry the residual error signal (**r** − **r**^*td*^) to the higher level corresponding to the prediction **r**^*td*^ from the higher level.

#### Network dynamics and learning rule

The dynamics, the learning rules and hence the above-mentioned architecture of a general PE module directly stem from probabilistic estimation methods. In the Bayesian framework, these originate from maximum a posteriori (MAP) approach. In this case, maximizing the posterior probability *P*(**r**, **r**^*td*^, *U*|**I**), which is equal to the product of generative models *P*(**I**|**r**, *U*), *P*(**r**^*td*^|**r**) and prior probabilities *P*(**r**) and *P*(*U*), with respect to **r** and *U* provides the dynamics and learning rule respectively. Equivalently, in the framework of information theory, the minimum description length (MDL) approach leads to the same results by minimizing the coding length *E*, which is equal to negative log of posterior probability defined above, with respect to **r** and *U* (for details, see [14] and supporting information of [22]).

By assuming the probability distributions *P*(**I**|**r**, *U*) and *P*(**r**^*td*^|**r**) as Gaussians of zero mean and variances *σ*^2^ and $\sigma_{td}^{2}$ respectively, the total coding length *E* can be written as,

$$E=\frac{1}{\sigma^{2}}{\left(I-Ur\right)}^{T}\left(I-Ur\right)+\frac{1}{\sigma_{td}^{2}}{\left(r-r^{td}\right)}^{T}\left(r-r^{td}\right)+g\left(r\right)+h\left(U\right)$$ (1)

here, *g*(**r**) and *h*(*U*) are the negative log of prior probabilities *P*(**r**) and *P*(*U*) respectively. Minimizing the coding length *E*, with respect to **r** (using the gradient descent method) provides the dynamics of PE module as,

$$\frac{dr}{dt}=-\frac{k_{1}}{2}\frac{\partial E}{\partial r}=\frac{k_{1}}{\sigma^{2}}U^{T}\left(I-Ur\right)+\frac{k_{1}}{\sigma_{td}^{2}}\left(r^{td}-r\right)-\frac{k_{1}}{2}g^{\prime }\left(r\right)$$ (2)

here, *k*_1_ is a rate parameter that governs the rate of descent towards a minimum of *E*, and *U*^*T*^ is the transpose to weight matrix *U*. The steady state of this dynamical equation provides an optimum-estimate, which is regarded as the representation of the input. Coding length *E*, roughly, can be seen as the mean square error at the input and the output level of a PE module, subjected to constraints of prior probabilities. And minimization of the coding length is equivalent to optimization of estimate by recurrently matching of estimate to the corresponding “sensory driven” input from lower area as well as “context driven” prediction signal from higher area. The prediction signal *U***r** is the linear combination of basis vectors *U*_*i*_’s. The *U*_*i*_ is the *i*^*th*^ column of the matrix *U*, and represents the receptive field for *i*^*th*^ neuron. The weighted coefficient in this combination, *r*_*i*_, represents the response of *i*^*th*^ neuron having receptive field *U*_*i*_. The visual representation of the prediction *U***r** corresponding to optimum-estimate **r** is, in this study, termed as “perceptual image.”

Furthermore, the minimization of coding length *E*, with respect to *U* using gradient descent method provides the learning rule for basis matrix *U* as,

$$\frac{dU}{dt}=-\frac{k_{2}}{2}\frac{\partial E}{\partial U}=\frac{k_{2}}{\sigma^{2}}\left(I-Ur\right)r^{T}-\frac{k_{2}}{2}h^{\prime }\left(U\right)$$ (3)

here *k*_2_ is learning rate, which operates on the slower time scale than the rate parameters *k*_1_, and **r**^*T*^ is the transpose of state vector **r**. This learning rule can be seen as of Hebbian type. In this study, prior probability, *P*(**r**), on state vector **r**, is chosen according to sparse coding; where it is assumed that the visual system encodes any incoming signal with a small set of neurons from the available larger pool [28]. The kurtotic prior distribution ($P\left(r_{i}\right)=exp\left(-\alpha log\left(1+r_{i}^{2}\right)\right)$) constrains the dynamics for the sparse representation of the input. This distribution gives us:

$$g^{\prime }\left(r_{i}\right)=2\alpha r_{i}/\left(1+r_{i}^{2}\right)$$ (4)

which is used in Eq (2). The prior probability distribution, *P*(*U*) has been chosen here to be Gaussian type, which finally gives us:

$$h^{\prime }\left(U\right)=2\lambda U$$ (5)

which is used in the Eq (3). Here *α* and *λ* are variance related parameters.

### Simulation

#### Network

**Fig 3:**
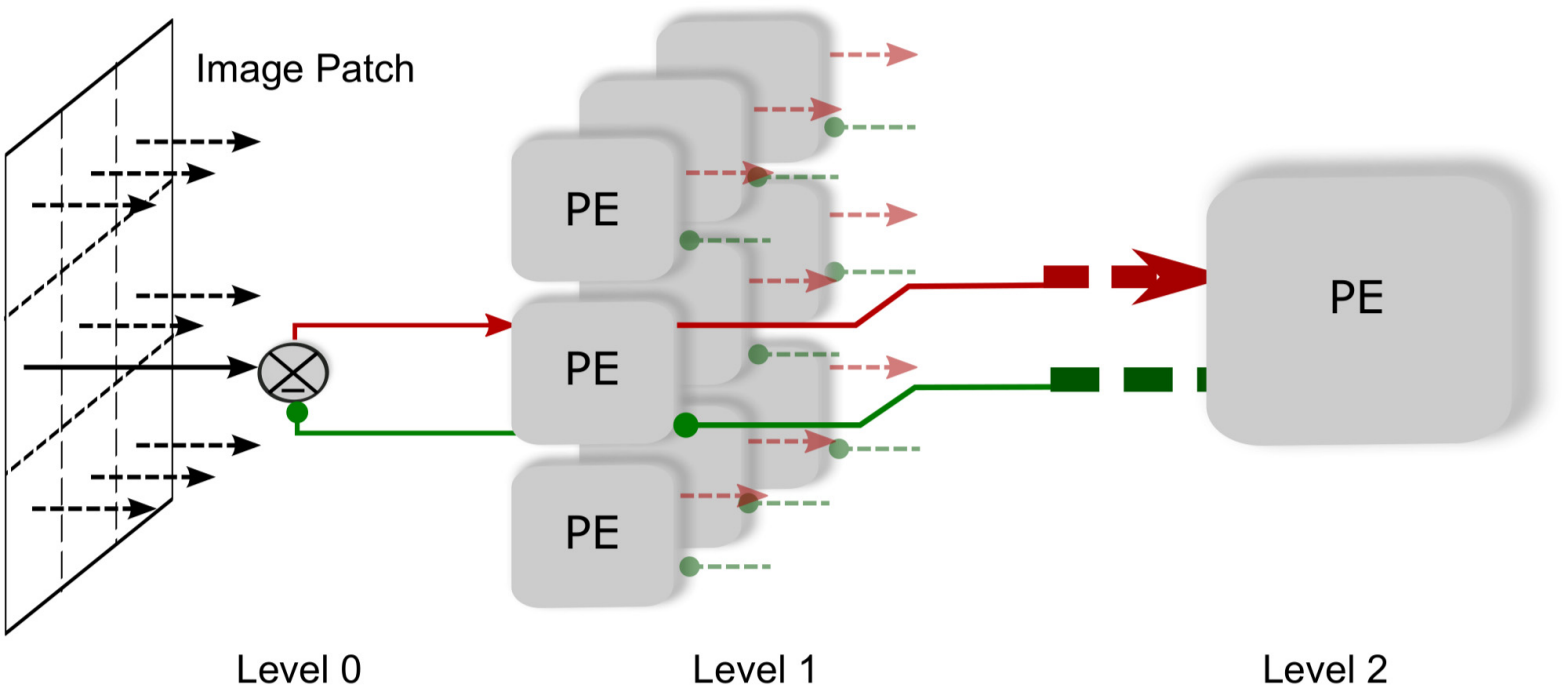
Three level HPC model network. Each of nine level 1 PE modules sends prediction to level 0 by feedback connection and receives error signal corresponding to their local image patches by a feed-forward connection. On the other hand, the PE module at level 2 sends the prediction signal to all level 1 modules and in reply, receives the error signal collectively from all these modules. Level 2, therefore, encodes larger visual patch and hence possess the larger receptive field.

In this work we simulated a three level linear hierarchical predictive network (See Fig 3). In this network, Level 1, which is equivalent to V1, consists of 9 PE modules. These modules receive input from level 0 and send the output to the solitary module at level 2. Level 0 is equivalent to the LGN and level 2 is equivalent to V2. Therefore, the PE module at level 2 receives input from all the nine level 1 PE modules and sends back the feedback signal to all of them. This architecture is based on the fact that the visual area higher in hierarchy operates on a higher spatial scale.

Each of nine PE modules at level 1 consists of 64 PE neurons, 144 Prediction carrying neurons, 64 afferent error carrying neurons and 64 error detecting neurons for conveying the residual error to level 2. The layer 2 module consists of 169 PE neurons, 576 prediction carrying neurons and 169 error carrying neurons.

#### Training

**Fig 4:**
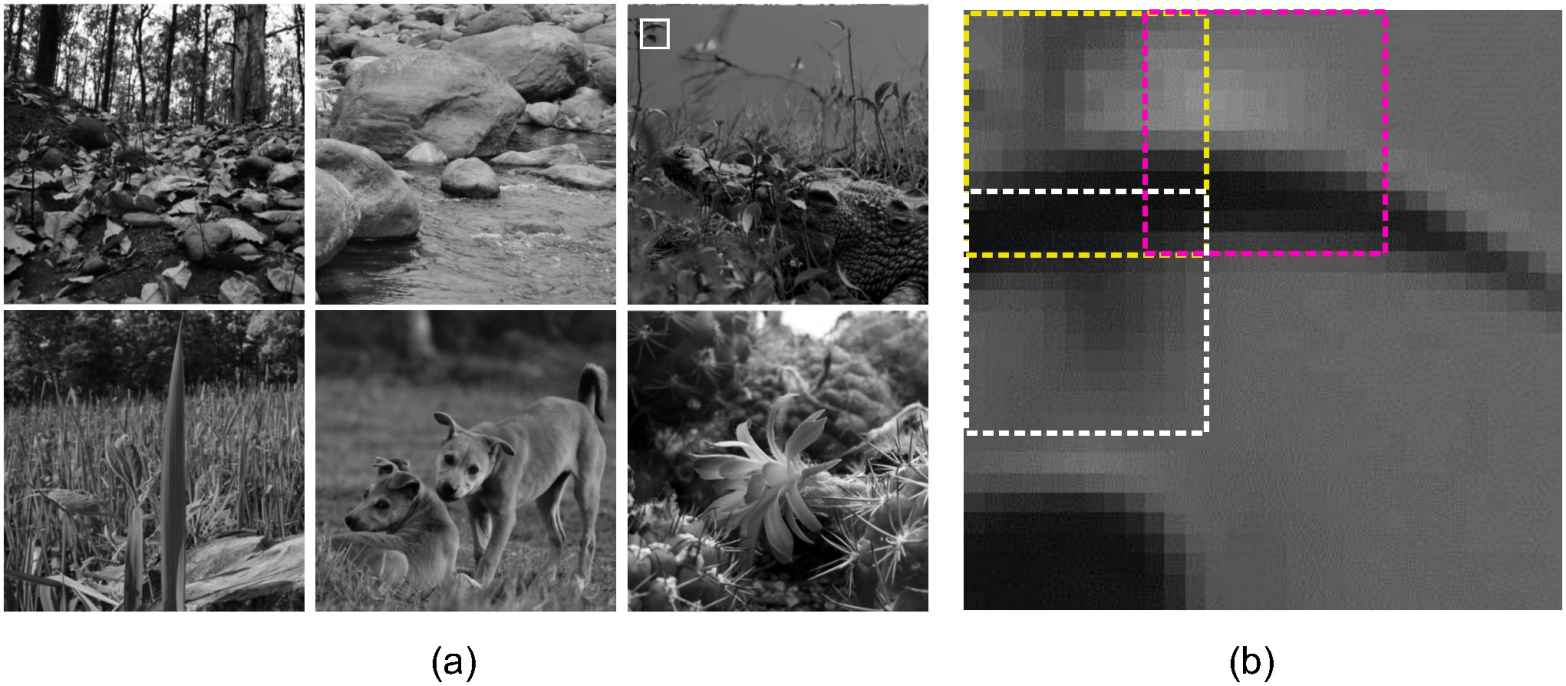
Natural images. a) These images, taken from of different natural environments, are used for simulation. b) A typical sample of 30 x 30 pixel image patches extracted from the natural image (top rightmost) from the position shown by the white rectangle. Each of these patches is broken down to 9 sub-patches of 12 × 12 pixel each with 3 overlapping pixels. Three such sub-patches are shown here by three dotted rectangles in yellow, magenta and white. Each of these sub-patches forms the local input to the 9, level 0 modules in the HPC model network.

Six natural images (Fig 4a) of size 512 × 512 pixels were used for training after pre-processing. The pre-processing involved DC removal and the filtering of images with circular symmetric whitening/lowpass filter with spatial frequency profile *W*(*f*) = *fexp*(−(*f*/*f*_0_)^4^)(see [28, 32]). Cutoff frequency *f*_0_ was taken to be 200 cycles/image. The pre-processing has been argued to emulate the filtering at LGN [33]. Variance normalized 1000 batches of 100 image patches of size 30 × 30 pixel, which were extracted from randomly selected locations from the randomly selected pre-processed images, were given as input to the network. A single 30 × 30-pixel image consisted of nine tiled 12 × 12-pixel image patches, which were overlapped by 3 pixels (see Fig 4b) and which were fed to the corresponding level 1 PE modules. For each batch of image patches, the network was allowed to achieve steady states (according to the Eq (2)) and the average of these states was used to update the efficacy of neurons (according to the Eq 3), initially assigned random values. During training, to prevent the efficacy vectors *U*_*i*_ (columns of *U* or rows of *U*^*T*^) from growing unbound, the gain (L2 norm), $l_{i}=\sqrt{U_{i}\cdot U_{i}}$, were adopted, as $l_{i}^{new}=l_{i}^{old}{\left(\langle r_{i}^{2}\rangle /\sigma_{goal}^{2}\right)}^{\gamma }$, so that the variance of *r*_*i*_ remain at appropriate level (for the details see [32]). Here $\sigma_{goal}^{2}$ is desired variance of *r*_*i*_ and *α* is gain adaption rate. The level 1 was trained first and then the level 2. Parameter values used in this study are: *k*_1_ = 1, *k*_2_ = 3, *σ*^2^ = 3, $\sigma_{td}^{2}=10$, *α* = 0.05 at level 1 and 0.1 at level 2, *λ* = 0.0025, $\sigma_{goal}^{2}=0.05$, *γ* = 0.02.

#### Blind spot implementation

To mimic the blind spot the feed-forward connection in a certain area was removed from the model network, which was pre-trained with usual feed-forward connections. The removal was implemented by setting the efficacy of early feed-forward (level 0–level 1) neurons, that carry the error signal corresponding to the middle region (of size 8 × 8) of input patches (of size 30 × 30), to zero. This “pre-training”, the training before the creation of the blind spot, captures the fact that the active neurons in deep layer (5/6) corresponding to filling-in has been reported to be of binocular type. These neurons were found to respond to the inputs from both eye and hence, possess binocular receptive field. Additionally, these neurons also exhibit greater sensitivity to the inputs from the other eye (non-BS eye) [6, 7]. It is, therefore, natural to assume that, in normal binocular vision the feed-forward input from non-BS eye will cause the receptive fields (of these deep layer neurons) to develop.

## Results

**Fig 5:**
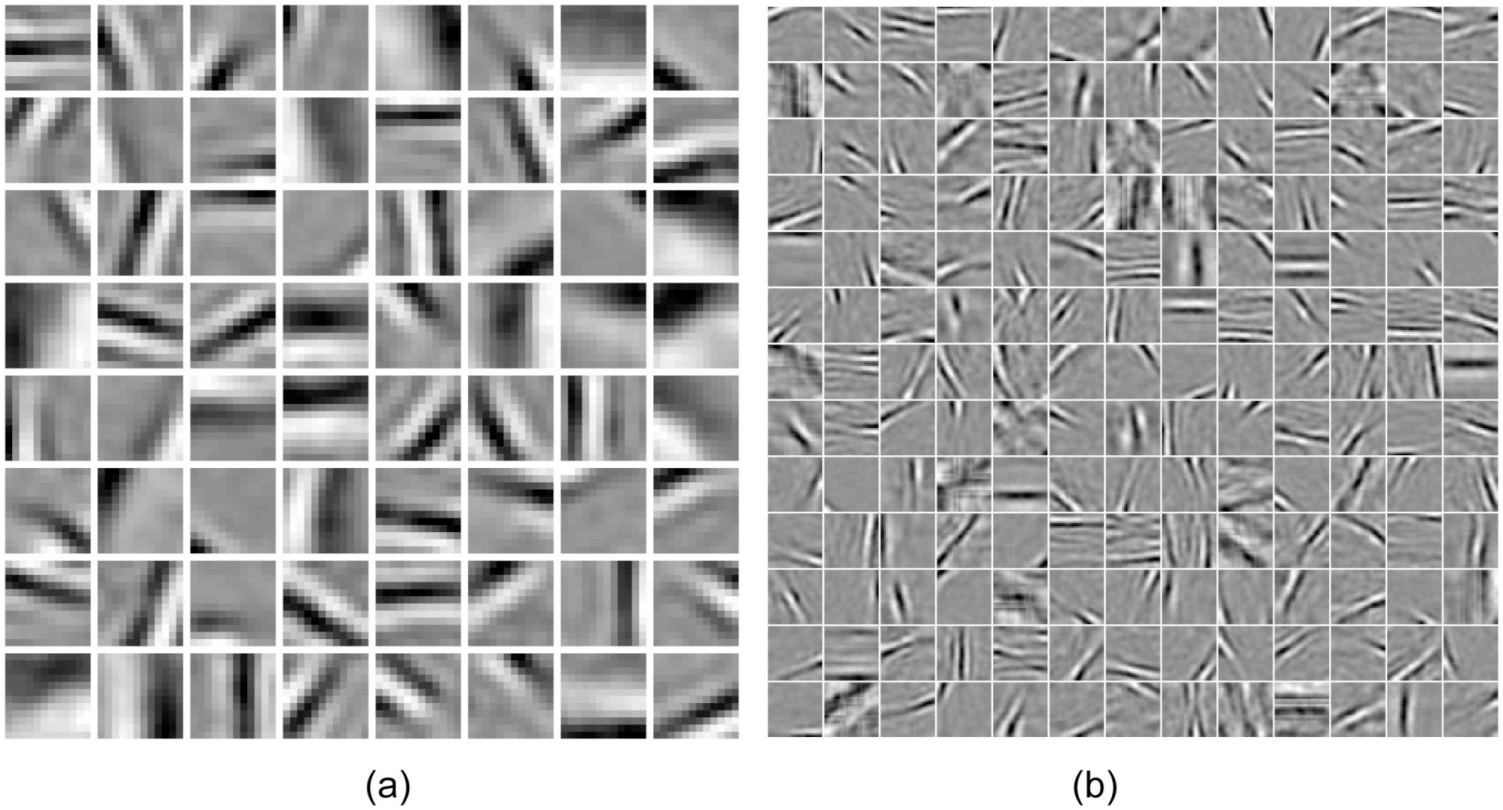
Learned synaptic weights. (a) Learned receptive field of 64 feed-forward neurons of the size 12 × 12 pixels of BS module at the level 1 and, (b) Learned receptive field 169 feed-forward neurons of the size 30 × 30 at the level 2.

To ascertain whether the computational mechanism of HPC could account for filling-in completion across the blind spot, we conducted a pair of experiments by stimulating the trained HPC model network with bar stimuli. The HPC network was allowed to learn the synaptic weight of model neurons, by exposing it to natural image patches under the constraints of the sparseness of model neuron responses (see method). The learned synaptic weights of neurons carrying feed-forward signal of one of the modules at level 1 and level 2 are shown in Fig 5. The weighting profiles at level 1 (Fig 5a) resemble the Gabor-like receptive field at V1, which is similar to the results reported earlier in several studies [14, 20, 28]. The weighting profile at the level 2 (Fig 5b) resembles the more abstract visual features: long bar, curve, etc. The blind spot was emulated in the network by removing feed-forward connection (see method), whereas, the training was performed on a network by keeping feed-forward connection intact. We designate the network with the blind spot as BS network and the one without the blind spot as a non-BS network.

### Filling-in of shifting bar

**Fig 6:**
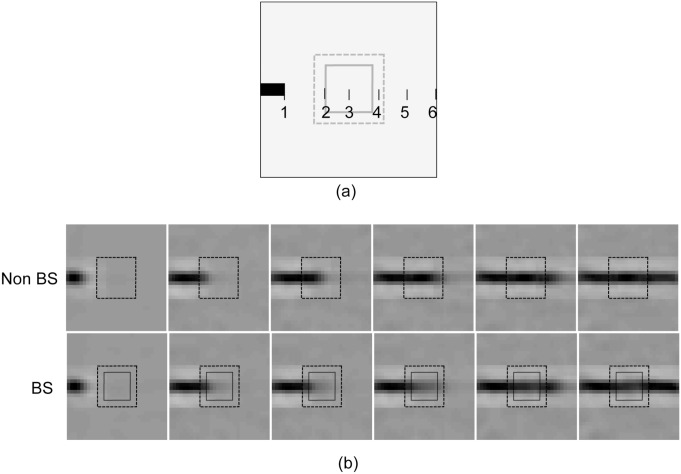
Shifting-bar. a) A typical 30 × 30 pixel stimulus is shown here. The darkened object in the stimulus is a bar, whose endpoint is represented by the number 1. Five more stimuli were constructed by shifting the bar end to positions 2 to 6. The larger rectangle of size 12 × 12 pixels (shown by the dotted line at the center) indicates the extension of BS module and the smaller one of size 8 × 8 (shown by the solid line) indicate the extension of blind spot. b) Generated 30 × 30 “perceptual images” corresponding to response profile of PE neurons at level 1 of the HPC network for non-BS (top row) and BS (bottom row) cases are shown.

Both BS and non-BS Network were exposed to images of a horizontal bar of different length. One end of the bar was fixed at a position outside of the blind spot, whereas, the position of other end was varied (by one pixel at each instant) across the blind spot. Images of the bar for six different end positions are shown in Fig 6a. The response vector, **r**, of PE neurons in the central module in the model network (let say BS module) at level 1 and the sole module at level 2 was recorded for the different end position of the bar.

**Fig 7:**
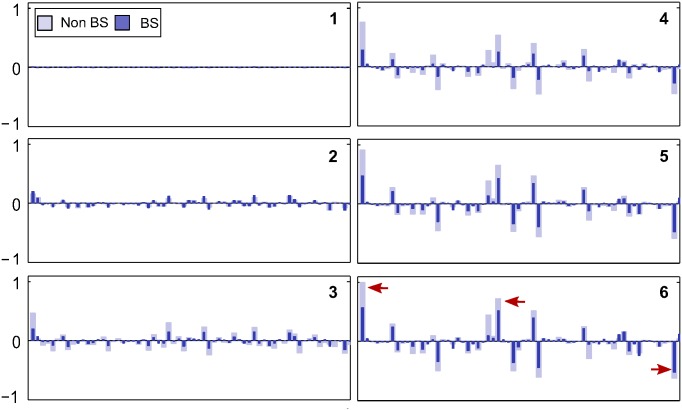
Responses profiles. Normalized responses of 64 PE neurons at BS module, corresponding to the six stimuli discussed in Fig 6a are presented. The dark blue bar represents the response of PE neurons for the BS network, whereas, the light blue bar represents the responses for the non-BS network. Three most highly active neurons (in bottom leftmost bar plot) are marked by red arrows.

**Fig 8:**
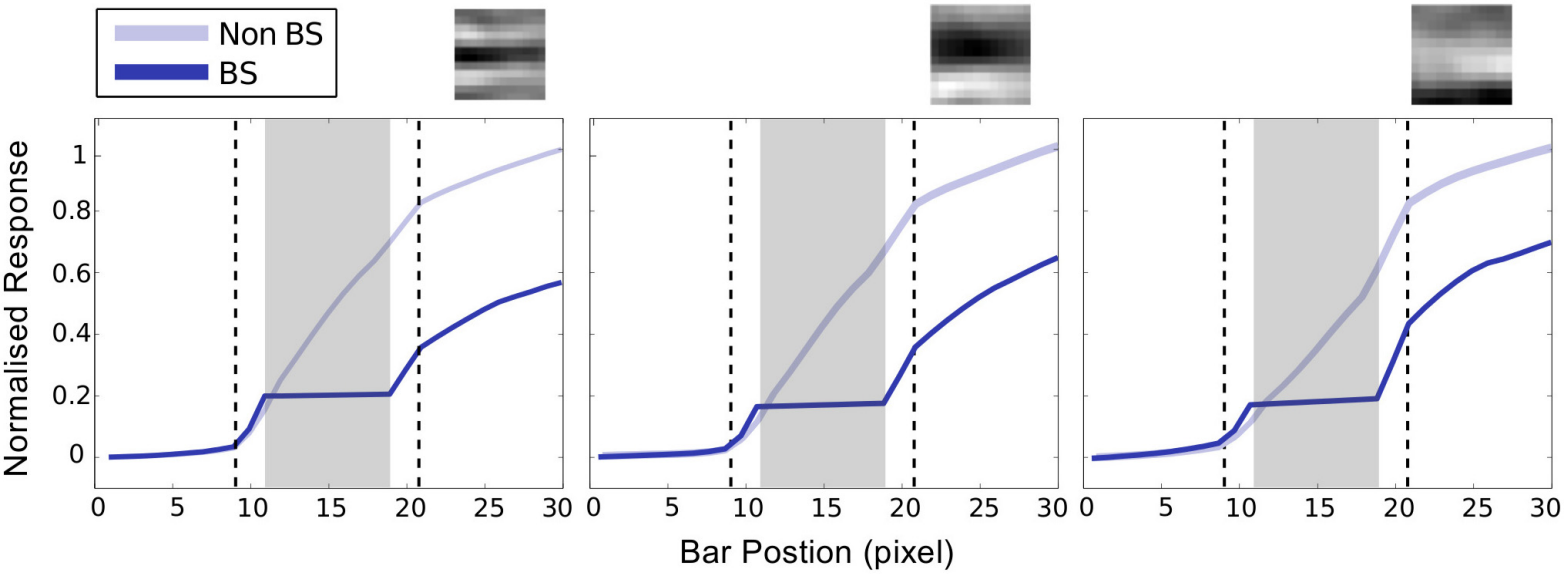
Response elevation in BS region at level 1. Plots of the absolute value of normalized response are shown against the bar position for three highly active neurons (indicated by red arrows in the sixth bar blot of Fig 7) In these plots, dotted rectangular area indicates the extension of BS module whereas, the solid gray rectangular area indicates the extension of blind spot. The receptive fields of these three neurons are shown at the top of the respective plots, which show that these neurons participated in encoding information of a horizontal bar. To compare the relative activity of the neurons we have plotted the absolute value of the responses instead of signed values of responses.

**Fig 9:**
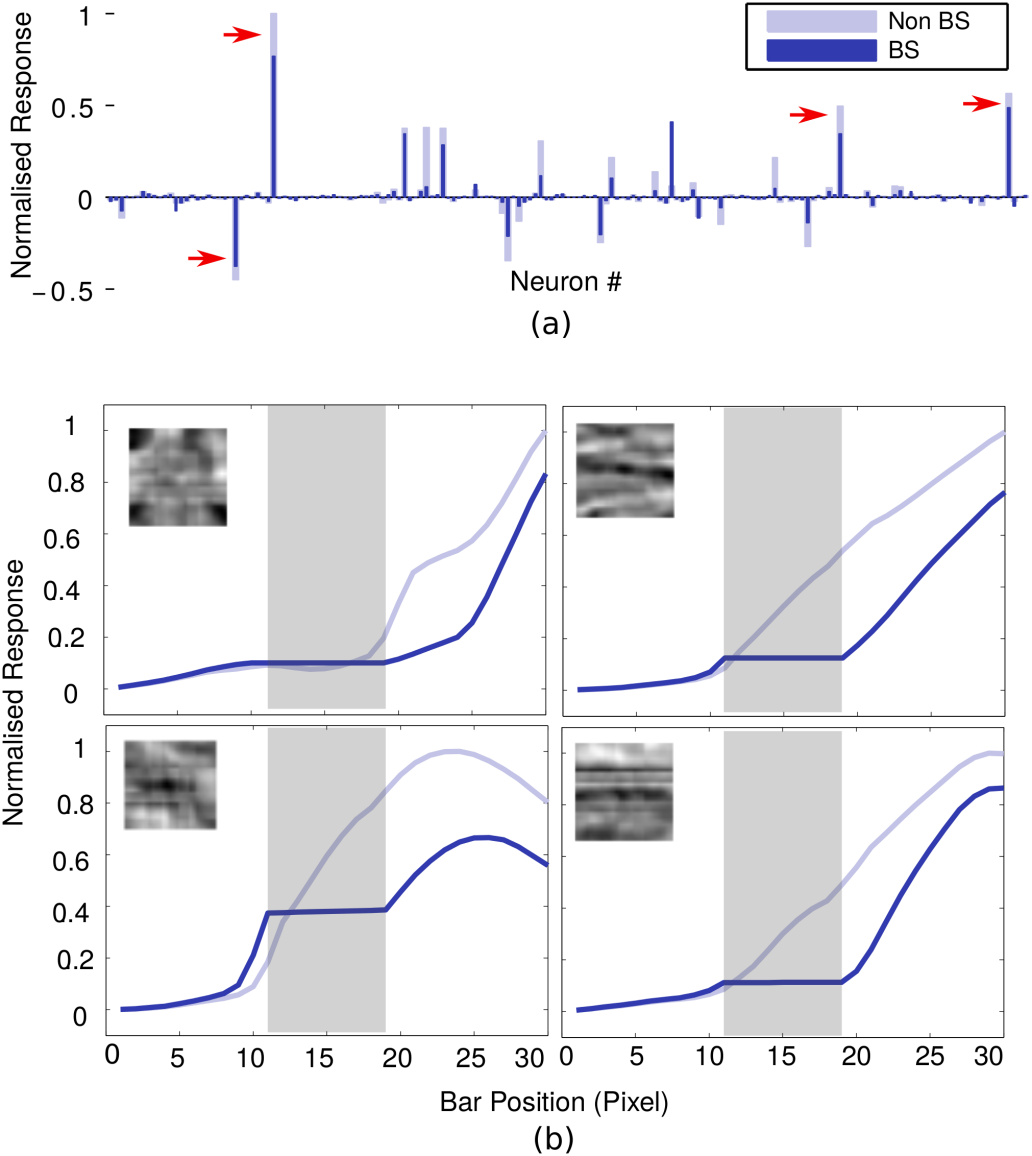
Response profile at level 2. (a) The normalized response of 169 neurons at level 2 corresponding to shifting bar stimuli for the end postilion 6. (b) Plots of the normalized absolute value of response of most active neurons at level 2 (marked as red arrow in (a)). The receptive field of these neurons is shown in the inset of their corresponding plots.

Fig 7 shows the bar plots of the response of 64 neurons in BS module at level 1 for six different bar position in both model networks(BS and non-BS). The comparison shows that almost the same set of a small number of neurons responded in both networks. The receptive field of the highly responsive neurons in this set possesses a horizontal bar-like structures. We plotted the response of some of these highly responsive neurons against the bar position (varying by one pixel) (see Fig 8) which show that these neurons exhibited elevated response when the varying end of bar crosses the blind spot. These elevated responses, in BS network, come reasonably close to the maximum response exhibit by these neurons in non-BS neuron. The closeness of responses indicates the representation of objects in the BS network is similar to the one in the non-BS network. This is reflected in the corresponding “perceptual images” (see Fig 6b) reconstructed using the generative process. The response profile of level 2 neurons is shown in Fig 9. The most active PE neurons at level 2 exhibit similar response as level 1 neurons and possess horizontal bar like receptive field, which is quite expected.

It is evident from these results that, in the case of BS Network, as long as the bar end reside inside the blind spot the response of neurons, in the BS module, remained constant and relatively low (Figs 7 and 8) which results in the perception of a bar of constant length on one side of the blind spot. On the other hand, when the bar crosses the blind spot, the responses are elevated significantly, and the filling-in completion occurred. These could also be understood by observing the relative deviation of the response of each neuron (typically the highly responsive neurons) in both networks (Fig 7) when bar end crosses the blind spot. These results are consistent with the findings of neurophysiological studies on macaque monkeys [7].

**Fig 10:**
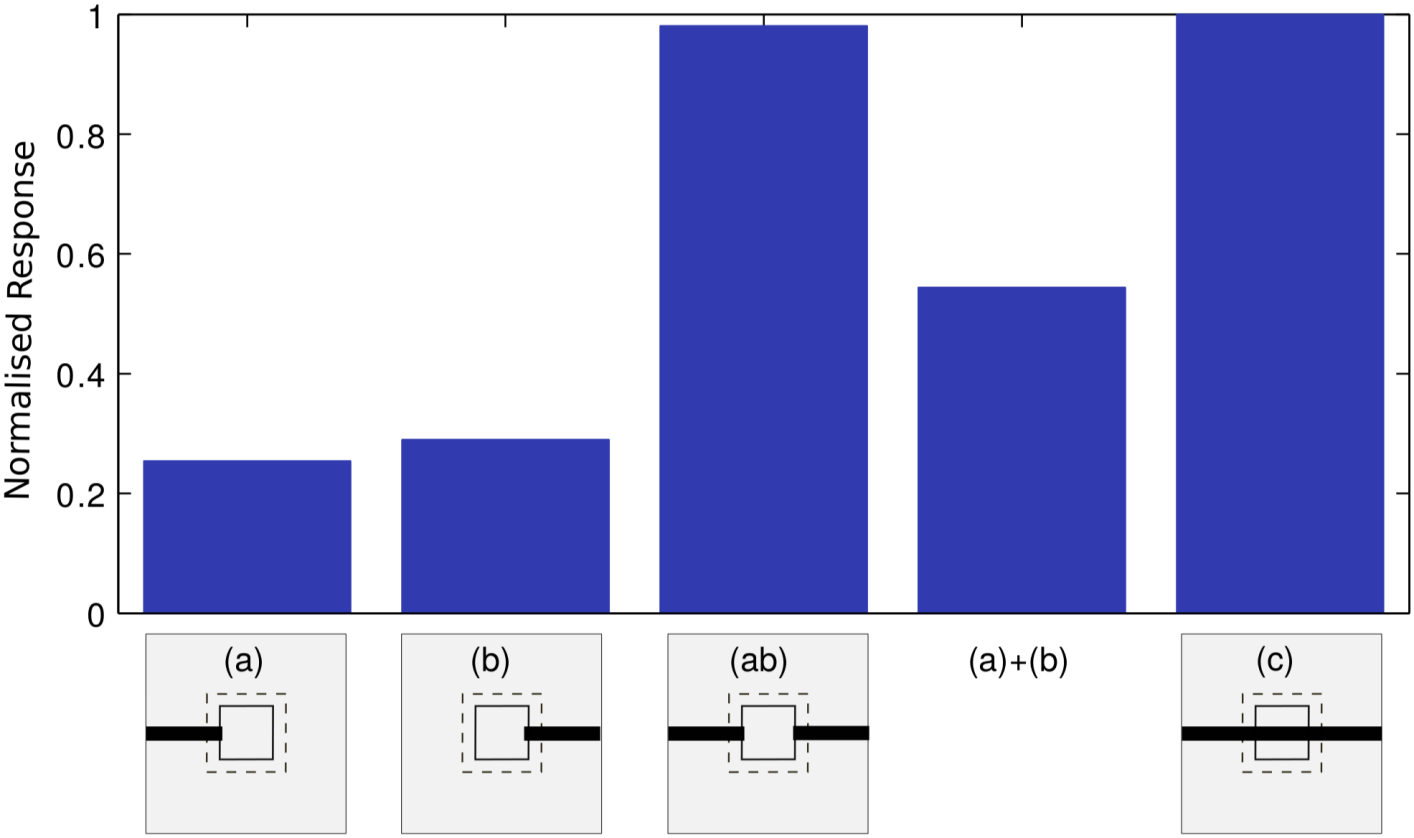
Nonlinearity in the response profile. Average normalized responses of neurons (in BS module) to various stimulus conditions are presented. Estimated responses to stimuli a, b, c and ab, as well as the sum of the responses to a and b are shown, where ab is a combination of stimuli a and b. Each stimulus is schematically shown below each bar plot, where each bar plot shows the mean of normalized responses of 8 most responsive neurons in the BS module. Conventions are same as shown in Fig 8.

It is evident from Fig 8 that when filling-in occur, the response profile changes abruptly in a non-linear fashion. In order to verify the explicit correlation between the non-linear response and the filling-in completion, the response of the most responsive neurons were examined by exposing the BS network with different stimulus combinations (a, b, c, ab and a+b) as described in Fig 10. The responses of these neurons to these four stimuli were compared. Stimuli are shown in the inset at the bottom of the figure and the corresponding responses are presented as bar plots above each stimulus. As shown in Fig 10, the response to ab stimulus is significantly larger than the sum of the responses to a and b (shown as a+b) even though stimuli a and b separately stimulated similar areas of the RF exposed ab stimulus. This indicates that the abrupt change in the magnitude of the response during filling-in completion can not be explained by the stimulation of the receptive field extending out from the opposite side of the blind spot. In other words, the abrupt response increase (non-linearity) is correlated with the perceptual completion and is not predictable from a simple summation rule.

### Filling-in for misaligned bars

**Fig 11:**
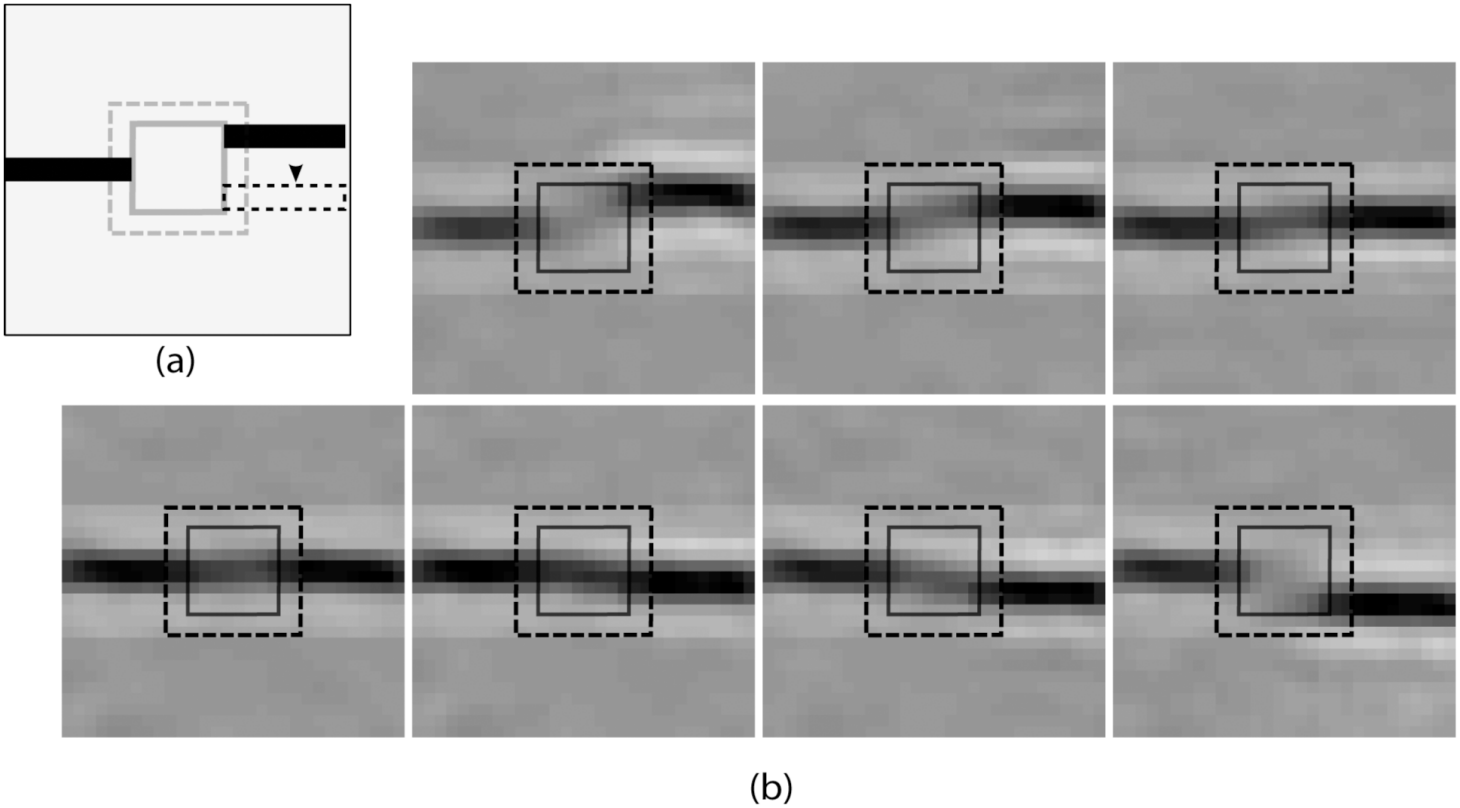
Nonaligned bar investigation. a) A typical stimulus is shown, where two non-aligned bar segments are presented at opposite sides of the blind spot. For our study, one bar was fixed (left one) and the other one was shifted vertically by one pixel per instant for the seven positions emulating seven stimuli. b) The generated “perceptual images” for those stimuli (as discussed in (a)) corresponding to the recorded response profile of PE neurons at level 1.

Two bar segments were presented on the opposite sides of the blind spot. One of these bar segments was kept fixed, whereas the other one was shifted along the vertical direction (see Fig 11a). We recorded the response of PE neurons, in the BS module, at Level 1 and generated the corresponding “perceptual images”. The result presented in the Fig 11b shows that the bar appears completed when both segments are aligned, but the filling-in fades away when misalignment increases. This result indicates that the filling-in completion is highly favorable for perfect alignment and have some degree of tolerance for misalignment. Similar results are reported in earlier psychophysical studies [30, 31].

To understand the mechanism of filling-in, we should recall that in HPC, feed-forward connection propagates up the residual error, corresponding to the current prediction made by higher area, for the betterment of the next prediction. The optimum-estimate, where prediction closely matches the “driving sensory” input as well as “contextual signal” from higher area, which produce a minimum prediction error, is then depicted as a percept of the input. However, the blind spot is characterized by the absence of such feed-forward connection. Therefore, the estimate made by higher areas prevails in the absence of error signal and this provides the ground for the filling-in completion at the blind spot.

Neurons at V2 learns the regular feature like long bar, curve etc. from the natural scene and operates on a larger area and context. Therefore, the initial estimate, which is based on these learned regular features, prevails and becomes an optimum-estimate for the inputs which only match with those regular features in the surrounding of the blind spot. This process initializes the filling-in at the V2. In the absence of feed-forward connection in BS region, the corresponding local optimum-estimate at level 1 will, therefore, evolve by matching the “context driven” feedback signal from level 2. This process at level 1 locally captures all the course of the completion process at level 2. Thus, the properties of the filling-in are highly determined by the matching of statistics of input stimuli around the blind spot and natural statistics learned by the network. Better the degree of matching, higher chances of completion.

For example, in the bar shifting experiment while the moving end of bar resides inside the blind spot, the incoming sensory input, which is of a short bar residing on one side of the blind spot, deviates reasonably from learned statistical regularity, in which the bars are usually longer (extended across the blind spot) [14]. That turns out as a non-completion of the bar and PE neurons at BS module, whose response represent bar, exhibit low response. On the other hand, when bar crosses the blind spot, the likelihood of matching of the input signal and the learned regular features suddenly increases and that leads to the abrupt elevation of the response of PE neurons which encodes the bar in BS module. This process resembles the AND-gate functionality and reflects the nonlinearity in response profiles shown above (Figs 8 and 10).

In the context of the second investigation, for the aligned bar segments, a continuous bar as an estimate is more likely in the landscape of set of similar features learned from natural scenes. This likelihood favors the completion of the aligned-bar segments. On the other hand, two non-aligned bar (non-aligned case in Fig 11a) is less likely to be part of a continuous object in the dictionary of learned feature set and that reflects in their non-completion. These suggest that the learned statistical regularity of natural objects along with the prediction-correction mechanism could play a significant role in filling-in completion.

We have presented some of the results in the form of “perceptual images”, which are generated from the activities of PE neurons, and did not associated these results to the visual angle, which is a common way of representing results in visual psychophysics. The reason to do so is, since the extension of receptive field and the blind spot are represented in terms of pixels, its relation to the actual shape and dimension of the blind spot (and receptive fields), and hence to the visual angle representation, is difficult to establish.

## Discussion

In this study, we have investigated the computational mechanism of filling-in at the blind spot. We postulated that this could be understood by examining the hierarchical predictive processing inside the visual cortex and therefore, conducted simulation studies on the three level HPC model network to investigate the filling-in completion using bar stimuli. The blind spot was emulated in the network by removing bottom-up feed-forward connection whereas; the training was performed on a network with usual top-down and bottom-up connections. In the first study, we recorded the response of PE neurons at V1 in BS module, while shifting a long bar across the blind spot, and additionally, generated the corresponding perceptual counterpart using the generative model. The non-linear response of the PE neurons, as well as the nature of filling-in of bar segments, was in agreement with experimental findings [7]. In another study, a pair of bar segments was presented on the opposite side of the blind spot, with varying alignment. We found that the filling-in completion, which occurs in the case of perfectly aligned bar segments, also occurs with a small degree of misalignment, but the degree of filling-in completion deteriorates with increasing misalignment. These results are also in good agreement with the physiological and psychophysical results reported earlier [7, 31].

Previous studies have suggested hierarchical predictive processing of natural images, as a general unified coding principle of visual system [14, 17, 18, 20, 34]. This study proposes that the same coding principle could also account for filling-in at the blind spot. Where, for an input stimulus around the blind spot, higher areas (V2) generates unified estimate (including the estimate corresponding to blind spot region) of the input stimuli on the basis of the learned statistical regularities of natural images. This estimate remains uncorrected due to the absence of error carrying feed-forward connection in BS region at V1 and therefore, local optimum-estimate is achieved essentially by top-down prediction. Influenced by learned statistical regularities, higher areas predicts a long continuous bar across the blind spot and this results in the perception of completion. The nonlinearity observed in the responses of PE neuron and, hence, the properties of filling-in, result from the degree of similarity between statistics of stimuli around the blind spot and the natural image statistics. This study provides another support to the suggestions of predictive coding as a general computational principle of visual cortex.

In some related studies [8, 10], the role of cortico-cortical (V2-V1) interaction in the filling-in of illusory contours and the surfaces was suggested. Neumann [9] proposed that the filling-in of illusory contour could be the outcome of modulation mechanism of feedback signal from V2, which enhance the favorable response profile of feature detecting neurons, mainly in the superficial layers, at V1, in the context of larger contour coded at V2. This model, therefore, has its limitation in explaining the completion across the blind spot where the activity is mainly found in the deep layer of the V1. In another recent study [35], authors tried to explain the non-linear behavior of neurons in filling-in in terms of interaction of top-down and bottom-up signal in a Bayesian framework, where the feed-forward signal carrying the “prediction match” plays a crucial role. However, in this study, we have demonstrated similar response profiles along with the other properties of filling-in under a simple, unifying framework of hierarchical predictive processing, where feed-forward signal carries the “prediction error” instead of “prediction match” which determines the activities of PE neurons. These PE neurons, in this framework, hypothetically reside in the deep layer of the cortex [16, 36], which is consistent with the physiological findings.

Regarding the “pre-training” mentioned in method section, one can argue that in the absence of feed-forward input, even the feedback signal from the higher area can possibly cause the associative receptive fields to develop, which could provide the basis for internal representation in the BS area. But this is not the case with neurons representing the blind spot in which, as we have already discussed, there seems to exist the feed-forward connection from the other eye (non-BS) in normal binocular vision to cause receptive fields of the deep layer neurons to develop. In this case, one can suggest that these neurons might get relatively reduce input strength in the BS region since these are getting input from only one eye rather than from both eyes and that can lead to different weighting profile. Without going into details of the integration of input, which can take the value of relative strength from half to one depending on assumption of integration (linear or nonlinear), we can discuss that even the reduction of input, in a reasonable amount, may not give rise to any qualitative change in the learned receptive fields of the neurons because the nature of the receptive fields is mainly governed by the statistical feature of the input. We, therefore, argue that this situation may not alter the generality of our approach.

In this work, we are suggesting the functional explanation of filling-in at the blind spot, which has been largely unexplained till this dates, under the simple linear predictive coding framework. Though this study provides some insight of this phenomenon qualitatively, a quantitative insight could be gained by incorporating a more detail HPC model. Some other recent studies [21, 23, 24] has expanded this framework from the standard one and has demonstrated that it can account for the various physiological and psychophysical results. With proper modification in line with the requirements of the blind spot, future studies in these detailed HPC frameworks could probably be useful for quantitative estimates of filling-in at the blind spot. Moreover, filling-in completion of a bar mainly governed by the the learned statistics of contrast information (edge, boundary, etc.) found in natural scenes. Positive outcomes of our investigation, therefore, suggest that surface filling-in at the blind spot could also be understood under the similar mechanism, given a proper surface representation in the hierarchical probabilistic framework, which is the present challenge of visual science. This study does not reject any possible role of intracortical interaction in V1 in filling-in completion. There could be some other (or more than one) prediction-correction pathway within V1, which can contribute to filling-in based on contextual information surrounding the blind spot.

In conclusion, recent studies on filling-in at the blind spot reveal that neural activities in BS area, in the deep layers of V1, are associated with filling-in completion. For example, in the shifting bar completion experiment, nonlinear neuronal activities are reported. Here, we have explained these activities in terms of hierarchical predictive coding principles of natural images and moreover, we have also demonstrated that these activities represent the filling-in. In the context of misaligned bars, perceptual results corroborate our findings, which show that the tolerance of filling-in varies with the alignment of the bars presented on opposite sides of the blind spot. All these results suggest that the filling-in could be a manifestation of a hierarchical predictive coding principle and, the nature of filling-in could be predominantly guided by the learned statistical regularities of the natural scene.
